# Human-specific remodeling of an endogenous retrovirus shapes structural diversity at acrocentric nucleolar organizer regions

**DOI:** 10.64898/2026.09.11.751073

**Published:** 2026-09-16

**Authors:** Mohammad Waseem, Amit Datta, Hudson O’Neill, Azait Imtiaz, Rafael Contreras-Galindo

## Abstract

Human acrocentric short arms harbor K111, an endogenous retrovirus that became an integral component of nucleolar organizer region (NOR)-associated architecture across all five acrocentric chromosome types. Here we combine complete human and primate genomes, haplotype-resolved population assemblies and parent-offspring trios to reconstruct its evolution and transmission. Predominantly full-length ancestral elements underwent human-specific expansion and remodeling, generating recurrent mosaics and individual-specific multicopy configurations. Population structural variation is concentrated in surrounding satellite landscapes rather than within K111-derived sequences themselves. Trio-resolved genomes reveal direct parental transmission, single-parent remodeling and, crucially, maternal-paternal remodeling in distinct configurations: large reciprocal domains with contrasting parental affinities and de novo sequence exchange within an offspring locus. Raw long reads support both maternal-paternal configurations. K111-derived loci associate with nucleolin-positive compartments. Together, these findings establish K111 as an evolutionarily dynamic component and molecular record of acrocentric NOR architecture, revealing unrecognized remodeling and sequence generation across human generations.

## Full Text Availability

The license terms selected by the author(s) for this preprint version do not permit archiving in PMC. The full text is available from the preprint server.

